# Supplementary material for: Factors Driving Individuals’ Attitudes toward Sugar and Sweet-Tasting Foods: An Analysis within the Scope of Theory of Planned Behavior
Source: Foods. 2024 Sep 28;13(19):3109. doi: 10.3390/foods13193109 (PMC11475485; doi:10.3390/foods13193109)
Supplement: Supplementary file 1 [file foods-13-03109-s001.zip › foods-3207802-supplementary.pdf]

Supplementary File S1

Questionnaire Used in the Study

Factors driving individuals' attitude toward sugar and sweet-tasting foods: An analysis within the scope of theory of planned behavior

1. I agree to participate in the study. *

Please mark only one option.

☐ Yes

☐ No

2. Gender

Please mark only one option.

☐ Male

☐ Woman

3. Age (Please write your birth year. For example, 1970

4. Weight (kg) (Please write as a whole number. For example, 85)

5. Height (cm) (Please write in cm, such as 182)

6. What is your educational background? *

Please mark only one option.

☐ Please mark only one option.

☐ Primary school-secondary school graduate

☐ Secondary education (high school) graduate

☐ Associate degree graduate

☐ Bachelor's degree

☐ Postgraduate (master's / doctorate / specialization)

7. What is your marital status?

Please mark only one option.

☐ Married

☐ Single

☐ Other:

8. Your profession?

Please mark only one option.

☐ Housewife

☐ Private sector employee

☐ Civil Servant

☐ Student

☐ Retired

☐ Freelance

☐ Unemployed / not working

☐ Employer

9. What is your monthly individual income?

Please mark only one option.

☐ Below minimum wage

☐ Around minimum wage

☐ 17.000-27.000 TL

☐ 27.000 - 37.000 TL

☐ 37.000 - 47.000 TL

☐ 47,000 TL and above

☐ I am a student, and I get pocket money from my family.

10. Do you smoke?

Please tick only one box.

☐ Yes

☐ No

11. Do you consume alcohol regularly?

Please tick only one box.

☐ Yes

☐ No

12. Do you exercise at least 150 minutes a week (such as brisk walking, running)?

Please tick only one box.

☐ Yes

☐ No

13. Which of the following describes your eating habits in the last 12 months? *

Please mark only one option.

☐ My eating habits have changed for the better

☐ My eating habits have changed negatively

☐ There was no change in my eating habits

14. Do you currently have a serious health problem that you feel is affecting your choices about eating and consuming food? (e.g. diabetes)

Please tick only one box.

☐ Yes

☐ No

15. If your answer is Yes, what is your disease that affects your choice of eating and consuming food (please specify)?

16. Do you have any food intolerances or allergies (especially sugar, sweeteners, wheat, gluten, grains and fruits)?

Please tick only one box.

☐ Yes

☐ No

17. If your answer is yes, what is it (please specify)?

18. Are you currently on any diet program? (e.g. restricting your diet to lose weight)

Please tick only one box.

- ☐ Yes
- ☐ No

19. If your answer is Yes, what is the purpose of your diet? (Please specify)

Consumption Survey

Please select how often you eat at least ONE portion of the following foods and drinks: (one portion includes: a piece of donut, a cookie, a scoop of ice cream, a glass of soda, etc.). You may need to swipe left and right to fill in on your phone.

Mark only one option on each line.

	I rarely or never consume	Less than 1 per week	1 time per week	2-3 times a week	4-6 times a week	1-2 times a day	3-4 times a day	More than 5 times a day
Biscuits (All types of sugary biscuits, cereal bars, gluten-free, biscuits)								
Breakfast cereals (ready-to-eat breakfast cereals, granola, muesli, porridge)								
Cakes (slices of cake, American muffins, croissants, pancakes, donuts, waffles)								
Chocolate and bars (All types of chocolate, chocolate bars)								
Dough desserts (such as baklava, revani.)								
Milk desserts (All milk desserts such as rice pudding, rice pudding, kazandibi, puddings)								
Sweet spreads and sauces (Chocolate spreads, peanut butter, flavored peanut butter, almond butter, cashew butter)								

Yoghurts (sweetened or non-diet)								
Yoghurts (unsweetened, sweetened or diet)								
Fruit juice and smoothies (unsweetened fresh fruit juice, fruit concentrate, sugar-free smoothies.)								
Non-alcoholic carbonated drinks (sugar-free or non-diet) (such as cola, fanta, sprite, soda, iced tea.)								
Non-alcoholic carbonated drinks (sugar-free or diet ones) (such as diet cola, zero.)								
Sugary tea, coffee, herbal teas								
Sweetened tea, coffee, herbal teas								
Tea, coffee, herbal teas with added honey								

Jam, honey, molasses, marmalade								
Other drinks (such as lemonade, compote juice.								
Other desserts (such as aşure, tahini halva, semolina halva)								

Beverage Consumption Survey - Short

1.For the past month, please indicate your intake for each type of drink, "how often" and "how much each time".

2.Please indicate how often you drank the following drinks, for example, if you drank 5 glasses of water a week, check 4-6 times a week.

3. Indicate the approximate amount of the drink you drank each time, for example, if you drank 1 glass of water each time, check 1 glass under "how much eachXXX time".

4. When trying to estimate your intake (i.e. water) throughout the day, think about the total amount you drink. For example, if you drink 3 times a day and each time you drink 1 glass of water, select "3 times a day" and check 1 glass of water under quantity.

1. In the last 1 month, how often did you drink **100% FRUIT JUICE?**
Check only one option.
- ☐ Never or less than 1 time per week
- ☐ 1 time a week
- ☐ 2-3 times a week
- ☐ 4-6 times a week
- ☐ 1 time a day
- ☐ 2 times a day
- ☐ 3 times a day or more

2. Which of the following amounts of **100% FRUIT JUICE** did you drink at one time according to the frequency you indicated above?
Check only one option.
- ☐ Never or less than 1 time per week
- ☐ Less than 130 mL (large tea glass)
- ☐ 200 mL (regular standard water glass or small can)
- ☐ 240 mL (cup)
- ☐ 330 mL (standard can)
- ☐ 500 mL (standard pet bottle)
- ☐ More than 500 mL

3. In the last 1 month, how often did you drink **SWEETENED FRUIT JUICES (LEMONADE, CAPPY, CAPPY, TAMEK, AROMA, COMPOTE WATER, COMPOTE, HOŞAF, ORALET, FRUIT COCKTAIL, etc.)?**
- ☐ Never or less than 1 time per week
- ☐ 1 time a week
- ☐ 2-3 times a week
- ☐ 4-6 times a week

☐ 1 time a day
☐ 2 times a day
☐ 3 times a day or more

4. Which of the following amounts of **SWEETENED FRUIT JUICES (LEMONADE, CAPPY, CAPPY, TAMEK, AROMA, COMPOTE WATER, COMPOTE, HOŞAF, ORALET, FRUIT COCKTAIL, etc.)** did you drink at one time according to the frequency you indicated above?
Check only one option.
☐ Never or less than 1 time per week
☐ Less than 130 mL (large tea glass)
☐ 200 mL (regular standard water glass or small can)
☐ 240 mL (cup)
☐ 330 mL (standard can)
☐ 500 mL (standard pet bottle)
☐ More than 500 mL

5. In the last 1 month, how often did you drink **SUGARY CARBONATED DRINKS (COLA, PEPSI FANTA, SPRITE, SODA, FRUIT SODAS etc.**)?
Check only one option.
☐ Never or less than 1 time per week
☐ 1 time a week
☐ 2-3 times a week
☐ 4-6 times a week
☐ 1 time a day
☐ 2 times a day
☐ 3 times a day or more

6. Which of the following amounts of **SUGARY CARBONATED DRINKS (COLA, PEPSI FANTA, SPRITE, SODA, FRUIT SODAS etc.)** did you drink at one time according to the frequency you indicated above?
Check only one option.
☐ Never or less than 1 time per week
☐ Less than 130 mL (large tea glass)
☐ 200 mL (regular standard water glass or small can)
☐ 240 mL (cup)
☐ 330 mL (standard can)
☐ 500 mL (standard pet bottle)
☐ More than 500 mL

7. In the last 1 month, how often did you drink **sugary energy and sports drinks (RED BULL, BURN, ROCKSTAR, GATORADE, POWERADE, etc.)?**
Check only one option.
☐ Never or less than 1 time per week
☐ 1 time a week
☐ 2-3 times a week
☐ 4-6 times a week
☐ 1 time a day
☐ 2 times a day
☐ 3 times a day or more

8. Which of the following amounts of **sugary energy and sports drinks (RED BULL, BURN, ROCKSTAR, GATORADE, POWERADE, etc.)** did you drink at one time according to the frequency you indicated above?
Check only one option.
☐ Never or less than 1 time per week
☐ Less than 130 mL (large tea glass)
☐ 200 mL (regular standard water glass or small can)
☐ 240 mL (cup)
☐ 330 mL (standard can)
☐ 500 mL (standard pet bottle)
☐ More than 500 mL

9. In the last 1 month, how often did you drink **DIET OR ARTIFICIALLY SWEETENED BEVERAGES (DIET COLA, MAX, ZERO, SUGAR-FREE SODA, SUGAR-FREE SPORTS DRINKS, SUGAR-FREE ENERGY DRINKS)?**
Check only one option.
☐ Never or less than 1 time per week
☐ 1 time a week
☐ 2-3 times a week
☐ 4-6 times a week
☐ 1 time a day
☐ 2 times a day
☐ 3 times a day or more

10. Which of the following amounts of **DIET OR ARTIFICIALLY SWEETENED BEVERAGES (DIET COLA, MAX, ZERO, SUGAR-FREE SODA, SUGAR-FREE SPORTS DRINKS, SUGAR-FREE ENERGY DRINKS)** did you drink at one time according to the frequency you indicated above?
Check only one option.
☐ Never or less than 1 time per week
☐ Less than 130 mL (large tea glass)
☐ 200 mL (regular standard water glass or small can)
☐ 240 mL (cup)
☐ 330 mL (standard can)

☐ 500 mL (standard pet bottle)
☐ More than 500 mL

11. In the last 1 month, how often did you drink **SWEET TEA (ICETEA-FUSETEA-NESTEA DİDİ)?**
    Check only one option.
    ☐ Never or less than 1 time per week
    ☐ 1 time a week
    ☐ 2-3 times a week
    ☐ 4-6 times a week
    ☐ 1 time a day
    ☐ 2 times a day
    ☐ 3 times a day or more

12. Which of the following amounts of **SWEET TEA (ICETEA-FUSETEA-NESTEA DİDİ)** did you drink at one time according to the frequency you indicated above?
    Check only one option.
    ☐ Never or less than 1 time per week
    ☐ Less than 130 mL (large tea glass)
    ☐ 200 mL (regular standard water glass or small can)
    ☐ 240 mL (cup)
    ☐ 330 mL (standard can)
    ☐ 500 mL (standard pet bottle)
    ☐ More than 500 mL

13. In the last 1 month, how often did you drink **BREWED OR BAGGED TEA (black, green and others)?**
    Check only one option.
    ☐ Never or less than 1 time per week
    ☐ 1 time a week
    ☐ 2-3 times a week
    ☐ 4-6 times a week
    ☐ 1 time a day
    ☐ 2 times a day
    ☐ 3 times a day or more

14. Which of the following amounts of **BREWED OR BAGGED TEA (black, green and others)** did you drink at one time according to the frequency you indicated above?
    Check only one option.
    ☐ Never or less than 1 time per week
    ☐ Less than 130 mL (large tea glass)
    ☐ 200 mL (regular standard water glass or small can)
    ☐ 240 mL (cup)
    ☐ 330 mL (standard can)

☐ 500 mL (standard pet bottle)
☐ More than 500 mL

15. If you drink **BREWED OR BAGGED TEA (BLACK, GREEN AND OTHERS),** which option below is appropriate? *
Check only one option.
☐ I didn't drink
☐ Sugar free
☐ Sugary
☐ Artificial sweeteners (aspartame, saccharin, stevia, etc.)
☐ Don't know

16. In the last 1 month, how often did you drink **COFFEE (FILTER, AMERICANO, INSTANT COFFEE, etc.)**?
Check only one option.
☐ Never or less than 1 time per week
☐ 1 time a week
☐ 2-3 times a week
☐ 4-6 times a week
☐ 1 time a day
☐ 2 times a day
☐ 3 times a day or more

17. Which of the following amounts of **COFFEE (FILTER, AMERICANO, INSTANT COFFEE, etc.)** did you drink at one time according to the frequency you indicated above?
Check only one option.
☐ Never or less than 1 time per week
☐ Less than 130 mL (large tea glass)
☐ 200 mL (regular standard water glass or small can)
☐ 240 mL (cup)
☐ 330 mL (standard can)
☐ 500 mL (standard pet bottle)
☐ More than 500 mL

18. If you drink **COFFEE (FILTER, AMERICANO, INSTANT COFFEE,** etc.) which option below is appropriate? *
Check only one option.
☐ I didn't drink
☐ Sugar free
☐ Sugary
☐ Artificial sweeteners (aspartame, saccharin, stevia, etc.)
☐ Don't know

Evaluation of Consumers' Attitudes and Preferences Towards Sugar Consumption

	**Strongly Disagree**	**Disagree**	**Not Sure**	**Agree**	**Strongly Agree**
consuming foods and drinks high in free sugar as part of my daily diet is something I automatically perform.					
consuming foods and drinks high in free sugar as part of my daily diet is something I perform without having to consciously remember.					
consuming foods and drinks high in free sugar as part of my daily diet is something I perform					

without thinking.					
consuming foods and drinks high in free sugar as part of my daily diet is something I start to perform before I realize I am performing it.					
most individuals who are significant to me would approve of me consuming foods and drinks high in free sugar as part of my daily diet.					
most individuals whose opinions I value believe that I should consume foods and drinks high					

in free sugar as part of my daily diet.					
most individuals who are significant to me are consuming foods and drinks high in free sugar as part of their daily diet.					
it is mostly up to me whether I consume foods and drinks high in free sugar as part of my daily diet.					
it would be possible for me to consume foods and drinks high in free sugar as part of my daily diet.					
I have complete					

control over whether I consume foods and drinks high in free sugar as part of my daily diet.					
if I wanted to, I could easily consume foods and drinks high in free sugar as part of my daily diet.					
I intend to consume foods and drinks high in free sugar as part of my daily diet in the next month.					
I expect to consume foods and drinks high in free sugar as part of my daily diet in the next month.					
it is likely that I will consume foods and					

drinks high in free sugar as part of my daily diet in the next month.					
I have difficulty starting tasks.					
I immediately perform my chores.					
I find it difficult to get down to work.					
I am always prepared.					
I frequently waste my time.					
I start tasks right away.					
I tend to postpone decisions.					
I like to get to work at once.					
I need a push to get started.					
I tend to carry out my plans.					
I tend to crave sweet foods.					

I tend to crave sugars.					
I tend to crave sweeteners (removed).					
I want to reduce my sweet food intake.					
the presence or absence of sweet foods in my diet influences my mood.					
the presence or absence of sugars in my diet influences my mood.					
the presence or absence of sweeteners in my diet influences my mood (removed).					
I feel indifferent toward sweet foods.					

the sweet taste is physically addictive.					
sugar is physically addictive.					
when I consume sugars, I balance out my diet through exercising and/or eating other healthy foods.					
when I consume sweeteners, I balance out my diet through exercising and/or eating other healthy foods.					
when I consume sweet foods, I balance out my diet through exercising and/or eating					

other healthy foods.					
my preference and/or intake of sugars depends on how much knowledge I have on them.					
my preference and/or intake of sweeteners depends on how much knowledge I have on them.					
I only consume sweet foods during special occasions.					
I only consume sugars during special occasions.					
I only consume sweeteners during special occasions.					
I categorize my sweet food intake into					

either “special” or “normal.”					
my health or body image will determine whether I modify my sugar intake or not.					
my health or body image will determine whether I modify my sweet food intake or not.					
my health or body image will determine whether I modify my sweetener intake or not.					
individuals who I am with (family, friends, and colleagues) influence my sweetener intake (removed).					

individuals are highly concerned about cutting down on sweet foods.					
individuals are highly concerned about cutting down on sugars.					
individuals are highly concerned about cutting down on sweeteners.					
sugar is not as bad as fat for your health (removed).					
adding sugar in food products is unnecessary.					
sweeteners are worse for your health than salt.					

sweeteners are physically addictive.					
sweeteners are not as bad as fat for your health (removed).					
adding sweeteners in food products is unnecessary.					
I feel guilty whenever I consume sweeteners.					
labels are misleading and deceptive.					
the food environment hinders me from reducing my sweetener intake.					
I know where to find credible information on sugars.					
I know where to find credible					

information on sweet foods.					
I know where to find credible information on sweeteners.					
If someone asks me, “what are sweeteners?”, I can explain.					
If someone asks me, “what is sugar?”, I can explain.					
I do not know whether to consume sugars or sweeteners (removed).					
I know how to replace sugars with sweeteners in cooking/baking.					
I know what strategies or policies have been implemented					

for reducing sugar intake in Turkiye.					
The desire or need for sweet food changes with age.					
The desire or need for sugar changes with age.					
The desire or need for sweeteners changes with age.					
Completely eliminating sugar from my diet is impossible (removed).					
Completely eliminating sweet food from my diet is impossible (removed).					
Consuming less sugary foods/drinks is					

a good thing for me.					
Consuming less sugary foods/drinks is a healthy thing for me.					
Consuming less sugary foods/drinks is something I enjoy.					
Consuming less sugary foods/drinks is something I effortlessly perform.					
Consuming less sugary foods/drinks is a delicious thing for me.					
Consuming less sugary foods/drinks is something that is valuable to me.					
